# Estimating the incidence and risk factors of postpartum hemorrhage from the national ENACT network

**DOI:** 10.21203/rs.3.rs-7992461/v1

**Published:** 2025-11-21

**Authors:** Malarkodi J. Samayamuthu, Olga Kravchenko, Wei-Hsuan Lo-Ciganic, Eugene M. Sadhu, Seonkyeong Yang, Vanathi Gopalakrishnan, Shyam Visweswaran

**Affiliations:** University of Pittsburgh; University of Pittsburgh; University of Pittsburgh; University of Pittsburgh; University of Pittsburgh; University of Pittsburgh; University of Pittsburgh

## Abstract

Aggregated counts from electronic health records (EHRs) provide a rich source of real-world data that can be leveraged to investigate critical medical conditions such as postpartum hemorrhage (PPH). We used the Evolve to Next-Gen Accrual to Clinical Trials (ENACT) network, a large, federated network of EHRs, and conducted repeated annual cross-sectional analyses on PPH incidence, risk factors, and maternal comorbidities in a large cohort of 591,375 delivery hospitalizations during the period 2005 to 2022. During this time, there was a statistically significant increasing trend in the incidence of PPH, which is consistent with previous studies. Native Hawaiian or Other Pacific Islander women had the highest incidence of PPH (12.14%), followed by Asian (11.04%) and Black or African American (9.36%) women. In PPH deliveries, the top-ranked risk factor was placenta previa or accreta (19.58%), the top-ranked comorbidity was primary cesarean delivery (41.08%), and the commonest cause was uterine atony (76.5%).

## Introduction

Health sciences research often relies on real-world data from various sources to generate evidence on patient health status and healthcare delivery. Electronic health records (EHRs), which record patient contacts, treatments, and clinical activities within healthcare systems, are a valuable source of realworld data and are increasingly used in epidemiological, clinical, and translational studies. Two primary approaches have emerged for assembling EHR data across multiple institutions: centralized and federated. In the centralized approach, EHR data from participating institutions are transferred to a central data repository, where the data are standardized and quality-controlled. This approach facilitates sophisticated analytics with individual-level data. However, there can be significant delays in data availability for analysis and potential scalability issues as data volume grows. Examples of research programs that use a centralized approach include the All of Us Research Program^1^ and the National COVID Cohort Collaborative (N3C) initiative^2^. In contrast to the centralized approach, the federated approach retains each participating institution’s data locally while harmonizing the data across institutions to a common standard. This approach provides access to a larger volume of data with shorter time lags and poses lower security risks to healthcare systems. However, accessing and aggregating data from multiple institutions can be time-consuming or require sophisticated technology to ensure consistency and accuracy. Research programs that use a federated approach include the National Patient-Centered Clinical Research Network (PCORnet)^3^, the Consortium for Clinical Characterization of COVID-19 by EHR (4CE)^4^ and the Evolve to Next-Gen Accrual of patients to Clinical Trials (ENACT) network^5,6^.

The ENACT network is unique because it enables users with no programming knowledge to query the data repositories in the network in an interactive fashion. The institutional EHR data repositories in the network use either the Informatics for Integrating Biology at the Bedside (i2b2) or the Observational Medical Outcomes Partnership (OMOP) data models, which are linked via the Shared Health Research Information Network (SHRINE) platform^7^. The network is a collaboration of 57 data-contributing institutions (ENACT sites) affiliated with academic medical research centers. The SHRINE platform facilitates execution of queries that are constructed using medical terms in key domains, including demographics, diagnoses, procedures, medications, laboratory test results, and visit characteristics. To maintain the currentness of data, each site in the network updates its repository at least once a month. While the SHRINE platform enables querying of all sites on the network, not all may respond to every query due to data repository downtime, network traffic, and other technical issues. Furthermore, the set of responding sites may vary with each query, especially when executing a sequence of queries. Additionally, to ensure patient privacy, SHRINE automatically obfuscates the results of queries. While the ENACT network has the potential to accelerate evidence generation from large-scale analysis of EHRs, the first step is to evaluate the alignment of evidence generated from the network with findings from prior research that used individual-level data.

We conducted this evaluation using a case study on postpartum hemorrhage (PPH), an obstetric emergency and a critical condition that significantly impacts maternal morbidity and mortality worldwide^8–11^. PPH is one of the top five causes of maternal mortality in both resource-abundant and resource-limited countries, although the absolute risk of death from PPH is substantially lower in developed countries. In the United States (US), the incidence of PPH has increased from 2.7% in 2000 to 4.3% in 2019, although maternal mortality from PPH has decreased since the 1980s, with a reduction to 1.7 deaths per 100,000 live births in 2009^11,12^. Despite this decline, in 2019, PPH accounted for 13.7% of pregnancy-related deaths in the US^13^. Our primary goal was to assess the consistency of evidence obtained from SHRINE-generated aggregated data with previously published research employing individual-level data.

The American College of Obstetricians and Gynecologists (ACOG) defines PPH as either cumulative blood loss of greater than or equal to 1000 mL or blood loss accompanied by signs or symptoms of hypovolemia within 24 hours of childbirth^14^. The criteria apply to both vaginal and cesarean deliveries. PPH is divided into subtypes based on symptom onset. Primary PPH is hemorrhage that occurs between the third stage of labor (i.e., delivery of the placenta) and 24 hours after fetal delivery, and secondary PPH is bleeding that occurs more than 24 hours after delivery and up to 12 weeks postpartum. The causes of PPH are summarized by the “Four Ts,” which include tone (e.g., uterine atony), trauma (e.g., laceration, rupture), tissue (e.g., retained products of conception), and thrombin (e.g., coagulopathies). Uterine atony has been identified as the most common cause, accounting for approximately 80% of PPH deliveries^15^. Treatment of PPH requires prompt recognition and management of the cause of hemorrhage, involving a well-coordinated team effort to address it. Surgical interventions, such as hysterectomy, are used only as a last resort. Key maternal risk factors for PPH include intrinsic factors such as placenta previa, polyhydramnios, multiple gestation, and uterine leiomyomas or factors related to circumstances around labor and delivery such as severe preeclampsia, chorioamnionitis or endometritis, prolonged labor, and prior cesarean^8,16^. Additionally, comorbidities and other clinical factors such as pregestational and gestational diabetes, asthma, chronic hypertension, antepartum hemorrhage, or abruption may further increase the risk of PPH^8^. Prevention of PPH includes identifying and managing risk factors and associated comorbidities, as well as active management of labor. Identifying the risk factors is crucial for targeted preventative measures and improving maternal health outcomes^17^.

We used the SHRINE platform to obtain a comprehensive set of counts related to hospitalized deliveries and PPH that enabled the estimation of the proportion of deliveries affected by PPH, risk factors, and associated comorbidities. A repeated annual cross-sectional analysis was conducted to examine trends in PPH incidence and the burden of risk factors and comorbidities among affected women. We hypothesized that insights from the ENACT network would be consistent with findings from prior research using individual-level data from the Agency for Healthcare Research and Quality’s (AHRQ’s) National (Nationwide) Inpatient Sample (NIS)^8^. Our study is vital for validating the ENACT network’s effectiveness in generating real-world data in health sciences, especially in critical conditions such as PPH.

## Results

### Study Population

Data for this study were obtained from the ENACT network, comprising counts of women hospitalized for delivery, PPH deliveries, risk factors, and comorbidities. The raw dataset comprised 591,375 unique delivery hospitalizations collected from 8 ENACT active sites from 2005 to 2022. Though there are more sites in the network, we restricted our analyses to data from those sites that returned results for most queries to minimize missingness in the dataset.

Table 1 shows the age and racial characteristics, risk factors for PPH, and maternal comorbidities of all, with PPH, and without PPH deliveries. Most of the deliveries occurred in women aged 18 to 54 years (92.09%), with nearly all PPH deliveries (98.73%) occurring within this age range. Due to querying constraints, the maternal age was the woman’s age at the time of querying the network, not the age at delivery. Since the age at the time of delivery was unavailable, the breakdown by age provides only an approximate estimate of the percentages of PPH occurring within age groups. Hence, our analyses did not use age breakdown data (see the Methods section). White women had the most deliveries, with 330,335 (55.87%) total deliveries and 24,965 (53.79%) PPH deliveries. Black or African American women had 57,175 (9.67%) total deliveries and 5,130 (11.05%) PPH deliveries.

### Incidence, Causes, and Interventions in Postpartum Hemorrhage

Table 1 shows the breakdown of PPH rates among racial groups, risk factors, and comorbidities. Among risk factors, women with prior cesarian (no placenta previa or accreta) had a PPH rate of 5.27%, while those with placenta previa or accreta had the highest rate of 19.58%. Women with severe eclampsia had a rate of 9.66%, women with chorioamnionitis or endometritis had a rate of 9.63%, women with polyhydramnios had a rate of 14.94%, and women with multiple gestation had a rate of 17.29%. Among comorbidities, the highest rate of PPH occurred in women with \primary cesarean (41.08%), and women with obesity (33.64%).

Table 2 summarizes the incidence of PPH across racial groups, the number of risk factors, and the number of comorbidities. The overall incidence of PPH was 4.46% from 2005 to 2022. PPH incidence varied by race, with the highest rates observed among Native Hawaiian or Other Pacific Islander (12.14%) and Asian (11.04%) women, corresponding to crude incidence rate ratios (IRRs) of 1.51 and 1.37, respectively, compared to White women (reference group, 8.03%). Women with at least one risk factor (11.37%, IRR = 1.95) had more than twice the PPH rate of those with none (reference group, 5.82%), and women with at least two risk factors (11.87%, IRR = 2.04) had the highest PPH rate. Similarly, the incidence of PPH was significantly higher among women with at least one comorbidity (9.13%, IRR = 1.45), and it was highest among those with two or more comorbidities (9.62%, IRR = 1.53) relative to those with none (6.29%). These findings demonstrate a clear increase in PPH risk as risk factors and comorbidities accumulate.

**Figure 1:**
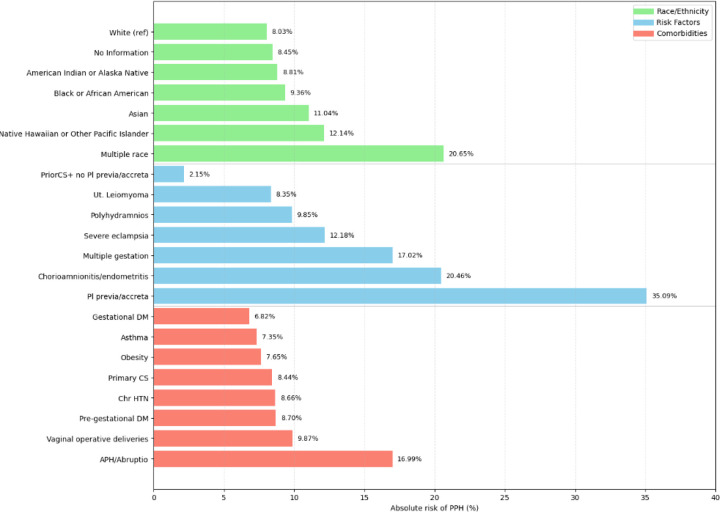
Incidence rates of postpartum hemorrhage (PPH). PPH rates (given as percentages) are ranked by race (green), risk factor (blue), and comorbidity (red).

Figure 1 shows the breakdown of PPH rates among racial groups, risk factors, and comorbidities.

Among risk factors, women with prior cesarian (no placenta previa or accreta) had the lowest PPH rate of 2.15%, while those with placenta previa or accreta had the highest rate of 35.09%. Women with severe eclampsia had a rate of 12.18%, women with chorioamnionitis or endometritis had a rate of 20.46%, women with polyhydramnios had a rate of 9.85%, and women with multiple gestation had a rate of 17.02%. Among comorbidities, the highest rate of PPH occurred in women with antepartum hemorrhage or abruption (16.99%) and in women with operative vaginal deliveries (9.87%).

Table 3 presents the causes of PPH and interventions. Among the causes, uterine atony was the commonest cause, occurring in 38,185 PPH deliveries, followed by tissue-related causes such as retained placenta, occurring in 15,515 PPH deliveries. Trauma was identified as the cause in 11,240 PPH deliveries, and thrombin-induced PPH occurred in 1,470 PPH deliveries. Among interventions, a surgical procedure was performed in 6,470 deliveries, of which 935 were hysterectomies. Manipulative procedures (e.g., manual removal of retained placenta or blood clots) were performed in 8,045 PPH deliveries, and blood transfusions were given in 3,030 PPH deliveries. The most common intervention was the administration of PPH-specific medications, which occurred in 24,080 deliveries.

### Trends in Postpartum Hemorrhage

**Figure 2:**
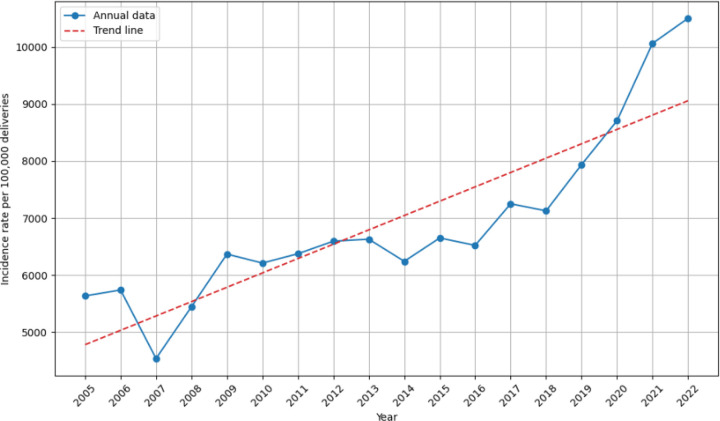
The trend in postpartum hemorrhage (PPH) incidence from 2005 to 2022. The incidence of PPH per 100,000 deliveries (blue) and the corresponding trend line (dashed red; Ptrend < 0.001).

Figure 2 shows the trend of PPH from 2005 to 2022. During this period, there was a statistically significant increasing trend, rising from 5,634 to 10,504 PPH per 100,000 deliveries (P_*trend*_ < 0.001). This trend persisted among women with one or more risk factors (P_*trend*_ < 0.001) and those with one or more comorbidities (P_*trend*_ < 0.001).

### Comorbidities in Postpartum Hemorrhage

**Figure 3:**
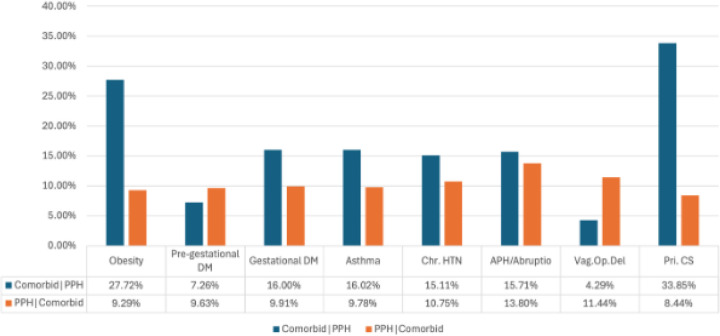
Comorbidity burden in postpartum hemorrhage (PPH). The prevalence of comorbidities in PPH deliveries (blue) and the reciprocal prevalence of PPH in comorbid conditions (orange).

Figure 3 illustrates the prevalence of various comorbidities in PPH deliveries and the reciprocal prevalence of PPH within those comorbid conditions. Comorbidities such as obesity, gestational diabetes, asthma, chronic hypertension, and antepartum hemorrhage or abruption have a high prevalence in PPH deliveries. The reciprocal prevalence of PPH is particularly high among women with antepartum hemorrhage or abruption (13.80%), operative vaginal deliveries (11.44%), and chronic hypertension (10.75%). While primary cesarean has the highest prevalence of PPH among comorbid conditions (33.85%), the prevalence of PPH in women undergoing primary cesarean is relatively lower at 8.44%. Conversely, obesity, with a high prevalence in PPH deliveries (27.72%), also shows a significant reciprocal PPH prevalence (9.29%).

## Discussion

The main objective of this study was to assess the trends in PPH, risk factors and comorbidities in the US using aggregated counts from the ENACT network. Our analysis revealed a rising trend in PPH, consistent with the trend observed in a previously published study covering the 2009–2019 period. This study relied on the NIS data, which comprises a 20% sample of all delivery hospitalizations in the US and is collected annually by the AHRQ’s Healthcare Cost and Utilization Project^8^. By including data from before 2009 and after 2019, our study corroborates previously observed trends and shows that the upward trajectory of PPH continues beyond the earlier study period. Given that PPH is life-threatening, our findings underscore the urgent need to identify women at risk of PPH for timely prevention and management.

Additionally, the study validates the utility of the data in the ENACT network for conducting research efficiently. Despite the data being available only as aggregate counts, we established reproducible data quality procedures and analysis workflows that confirm the reliability of the data in the network for similar analyses. Our querying methods were refined iteratively after discussions with the ENACT technical team to ensure data accuracy. This validation is significant because it demonstrates that the network’s data, when processed using rigorous data quality processes, can be a valuable resource for future research.

Based on the incidence rates of PPH across the various races in our results, we found that the Native Hawaiian or Other Pacific Islander women had the highest incidence of PPH (~ 12.1%), followed by Asian women (~ 11%), Black or African American women (~ 9.4%), and American Indian or Alaska Native women (~ 8.8%),. Our findings are consistent with prior studies that confirmed similar disparities using more rigorous statistical methods applied to individual-level EHR data and medical charts^18–20^. Our analyses identified the commonest risk factor as placenta previa or accreta (19.6% in PPH) and the commonest comorbidity as antepartum hemorrhage or abruption (19.1% in PPH). In concordance with the results obtained from AHRQ’s NIS data^8^, our results show that uterine atony is the commonest cause of PPH.

Overall, our analysis yields actionable findings regarding racial disparities, showing that Native Hawaiian or Other Pacific Islander, Asian, American Indian or Alaska Native, and Black or African American women have an increasing prevalence of PPH. The rank ordering of comorbidities and risk factors also emphasizes the importance of identifying and managing multiple gestations, prior cesarean, gestational diabetes, and chronic hypertension. These comorbidities and risk factors can help to inform future studies in designing personalized risk prediction models for PPH.

Our study has several limitations. First, to preserve patient privacy, the patient counts provided in SHRINE are obfuscated. This obfuscation may introduce some level of inaccuracy in the data, potentially affecting the precision of the analysis results. Because the counts for each query were generally large (in the tens or hundreds of thousands or millions), obfuscation likely did not affect the validity of the results.

Second, the technology used in ENACT calculates the number of unique patients meeting query criteria, which may lead to undercounting if multiple occurrences of the same diagnostic code are present. For instance, a count from a query may not account for second, third, or subsequent deliveries in the same woman, which may affect the accuracy of the trends and outcomes related to multiple deliveries. Depending on the research question, this can be mitigated by choosing an appropriate timeframe for querying the condition of interest.

Third, the diagnosis of PPH was based only on ICD-9-CM and ICD-10-CM codes for diagnosis, which is similar to prior research that used NIS data, to which we compared our results. Including indicators of blood loss, such as changes in hematocrit values or estimated blood loss measurements from the magnitude of the drop in hematocrit values, could potentially improve the accuracy of identifying PPH in EHR data. However, due to the limitations of the SHRINE platform, we were unable to obtain actual hematocrit values, changes in hematocrit values, or vital signs.

Fourth, we utilized only ICD-9-CM and ICD-10-CM codes for procedures, based on methodologies established in previous studies. Typically, all sites map their procedures to Current Procedural Terminology (CPT-4) codes, but not necessarily to ICD codes. The exclusion of CPT-4 codes may lead to incomplete data capture, potentially missing some delivery procedures.

Finally, due to the lack of individual-level data, we could not study women longitudinally, infer temporal relationships, or identify confounding factors to infer causality. Using aggregated count data to evaluate the correlation between PPH, risk factors, and comorbidities does not establish causal relationships. Additionally, drawing race-based conclusions is challenging since the counts of minority groups (e.g., American Indian or Alaska Native, Native Hawaiian or Other Pacific Islander) are small.

This case study illustrates the use of aggregated counts from EHRs from a federated network of sites across the US to assess trends in PPH among racial and age groups. We found a clearly increasing trend in PPH incidence from 2005 to 2022, supported by prior research. Further, aggregated counts were adequate to assess maternal risk factors and comorbidities incidence rates, and the findings were concordant with prior research. Data in the ENACT network is updated regularly, which provides the ability to rapidly obtain data for assessing recent trends in critical conditions such as PPH. Our finding underscores the need for better approaches to more accurately identify women at an elevated risk for PPH, while accounting for diverse racial groups. In the future, the limitations of aggregated data will be overcome by conducting studies on cohorts of patients with individual-level data (e.g., the global maternal and newborn health eCohorts^21^) that will yield more precise data to assess trends in PPH and associated risk factors and comorbidities.

## Methods

### Data Sources

We used SHRINE to query the ENACT network to obtain patient counts. The EHR data in the network is de-identified to ensure patient privacy and comply with Health Insurance Portability and Accountability (HIPAA) regulations, and SHRINE returns aggregate counts of patients meeting query criteria. To ensure privacy, SHRINE automatically obfuscates a patient count that a query returns by adding or subtracting a number up to 10 and rounds the resulting count to the nearest multiple of five^22^. Due to obfuscation, the same query may return slightly different counts when rerun, though these variations are typically negligible, especially when the counts are high. The University of Pittsburgh’s Institutional Review Board (IRB) approved using the ENACT network for a wide range of research under protocol number STUDY19080059.

The queries were submitted to all sites on the ENACT network, resulting in adequate responses from 8 active sites; counts obtained from these 8 sites comprised the raw dataset. To qualify for inclusion in the analyses, sites were required to meet specific inclusion criteria, ensuring data quality and completeness. Sites were required to have 20% or less missing annual counts or counts that are at least an order of magnitude lower than adjacent years, allowing for up to two years of imputed counts within the eighteen years from 2005 to 2022 for each query. A considerable reduction in count from one year to the next indicated potential data errors or the absence of relevant EHRs.

### Study Design and Data Extraction

We conducted repeated annual cross-sectional analyses using counts aggregated across all selected sites from 2005 to 2022. First, we identified women with delivery hospitalizations each calendar year using the International Classification of Diseases, Ninth and Tenth Revisions, Clinical Modification (ICD9-CM and ICD-10-CM) diagnosis and procedure codes (see Supplementary information). Next, within this cohort, we identified deliveries with PPH using ICD-9-CM and ICD-10-CM codes (see Supplementary information). Women with delivery hospitalizations without PPH served as a comparator group. We opted to rely solely on ICD codes for the diagnosis of PPH for several reasons, including prior research that used NIS data to which we compared our results relied on ICD codes, limitations of the SHRINE platform (which did not allow for calculating estimated blood loss by assessing the magnitude of the drop in hematocrit values), and ICD codes have been reported to have high specificity for PPH^23,24^. We executed two sequences of queries: one for all delivery hospitalizations and another for delivery hospitalizations with PPH.

### Outcomes

Our primary outcome of interest was the annual incidence rate of PPH among delivery hospitalizations, focusing on examining trends in incidence rates over the entire study period. Because of the potential limitations of obfuscated data, we compared demographics, risk factors, and comorbidities between the two groups: deliveries with PPH and deliveries without PPH. The demographics included women 15–54 years old stratified by 10-year age intervals and race (American Indian or Alaska Native, Asian, Black or African American, multiple races, Native Hawaiian or Other Pacific Islander, unknown, and White). Due to constraints in constructing queries in SHRINE, obtaining an individual’s age at a specific moment in the past was not possible. Thus, the maternal age we obtained was the woman’s age at the time of querying the network, not the age at delivery. Therefore, maternal age was not considered as a variable in our analyses.

We assessed several maternal risk factors, including prior cesarean (no placenta previa or accreta), placenta previa or accreta, severe preeclampsia, polyhydramnios, chorioamnionitis or endometritis, multiple gestation, and uterine leiomyomas. In addition, we assessed several comorbidities and other clinical conditions, including obesity, pregestational diabetes, gestational diabetes, asthma, chronic hypertension, antepartum hemorrhage or abruption, operative vaginal delivery, and primary cesarean (or first cesarean delivery). We categorized the causes of PPH according to the “Four Ts” (i.e., tone, trauma, tissue, thrombin) as secondary outcomes. Furthermore, we assessed surgical and medical PPH interventions, such as surgical procedures, including hysterectomy, manipulative procedures, blood transfusion, and medications (see Supplementary information). The PPH incidence, risk factors, and comorbidities were reported as counts and proportions.

### Data Analyses

We imputed missing counts using linear regression when there were two or more consecutive missing years or for a boundary year. For single-year missing counts, the counts for the preceding and subsequent years were averaged.

The trends in PPH incidence rates were examined within subgroups defined by race, presence of maternal risk factors, and presence of comorbidities. To assess the significance of the trend in PPH incidence over time, we employed the non-parametric Mann-Kendall trend test^25^. The Mann-Kendall test was chosen for its robustness against data irregularities and its ability to analyze trends without assuming a specific data distribution. Further, we assessed the incidence of PPH in each risk factor and comorbidity subgroup.

We computed the prevalence of comorbidities for women who had PPH deliveries (comorbidity | PPH), as well as the reciprocal prevalence of PPH delivery given these comorbidities (PPH | comorbidity). Data analyses were conducted using Microsoft Excel version 4.3.2 and Python version 3.7.3.

## Supplementary Material

Supplementary Files

This is a list of supplementary files associated with this preprint. Click to download.

- supplementaryinformation.docx

## Figures and Tables

**Table 1:** Demographic characteristics, risk factors, and comorbidities of all, with postpartum hemorrhage (PPH), and without PPH delivery groups. The percentages presented for maternal race, risk factors, and comorbidities are “column” percentages, which denote

Age* (unique patients)	All (≥1 delivery) n = 591,375	With PPH (≥1 event) n = 46,410	Without PPH n = 544,965	
< 18 years	33,420	20	33,400	
≥18 years	557,745	46,405	511,340	
18–54 years	544,610	45,820	498,798	
> 55 years	13,140	820	12,320	
Maternal race (unique patients)	All (≥1 delivery), n = 591,375	With PPH (≥1 event), n = 46,410	Without PPH n = 544,965	P-value
American Indian or Alaska Native	2,895 (0.49)	245 (0.53)	2,650 (0.49)	0.057
Asian	28,340 (4.79)	3,045 (6.56)	25295 (4.65)	< 0.0001
Black or African American	57,175 (9.67)	5,130 (11.05)	52045 (9.55)	< 0.0001
Multiple Races	460 (0.08)	90 (0.19)	370 (0.07)	< 0.0001
Native Hawaiian or Other Pacific Islander	1,400 (0.24)	150 (0.32)	1250 (0.23)	< 0.0001
Unknown	47,590 (8.05)	3,640 (7.84)	43950 (8.07)	0.021
White	330,335 (55.87)	24,965 (53.79)	305370 (56.06)	< 0.0001
Risk factors (unique deliveries)	All n = 858,404	With PPH n = 38,270	Without PPH n = 820,134	P-value
Prior cesarean (no placenta previa or accreta)	15,540 (1.81)	2,015 (5.27)	13,525 (1.65)	< 0.0001
Placenta previa or accreta	44,085 (5.14)	7,495 (19.58)	36,590 (4.46)	< 0.0001
Severe eclampsia	22,505 (2.62)	3,695 (9.66)	18,810 (2.29)	< 0.0001
Polyhydramnios	17,825 (2.08)	2,460 (6.43)	15,365 (1.87)	< 0.0001
Chorioamnionitis or endometritis	37,890 (4.41)	6,615 (17.29)	31,275 (3.81)	< 0.0001
Multiple gestation	29,430 (3.43)	3,775 (9.86)	25,655 (3.13)	< 0.0001
Uterine leiomyomas	27,770 (3.24)	2,995 (7.83)	24,775 (3.02)	< 0.0001
Comorbidities (unique deliveries)	All n = 858,404	With PPH n = 38,270	Without PPH n = 820,134	P-value
Obesity	138,650 (16.15)	12,875 (33.64)	125,775 (15.34)	< 0.0001
Pregestational diabetes	35,050 (4.08)	3,375 (8.82)	31,675 (3.86)	< 0.0001
Gestational diabetes	75,005 (8.74)	7,430 (19.41)	67,575 (8.24)	< 0.0001
Asthma	76,085 (8.86)	7,440 (19.44)	68,645 (8.37)	< 0.0001
Chronic hypertension	65,265 (7.60)	7,015 (18.33)	58,250 (7.10)	< 0.0001
Antepartum hemorrhage or abruption	52,845 (6.16)	7,295 (19.06)	45,550 (5.55)	< 0.0001
Operative vaginal delivery	17,445 (2.03)	1,995 (5.21)	15,450 (1.88)	< 0.0001
Primary cesarean	186,270 (21.70)	15,720 (41.08)	170,550 (20.80)	< 0.0001

**Table 2:** Crude incidence rates and incidence rate ratios (IRRs) of postpartum hemorrhage (PPH) deliveries by race, number of risk factors, and number of comorbidities. Absolute risk (crude incidence rate) is calculated as the proportion of PPH deliveries within each subgroup. Crude IRRs are presented relative to the reference groups: White women (race), those with no PPH risk factors, and those with no comorbidities.

			Absolute risk for each subgroup (crude incidence rate, %)	Crude incidence rate ratio (IRR)
Maternal race	All n = 858,404	With PPH n = 38,270		
All deliveries	858,404	38,270	4.46	
American Indian or Alaska Native	2,895	255	8.81	1.10
Asian	28,340	3130	11.04	1.37
Black or African American	57,175	5350	9.36	1.16
Multiple Races	460	95	20.65	2.57
Native Hawaiian or Other Pacific Islander	1,400	170	12.14	1.51
Unknown	47,590	4020	8.45	1.05
White (Reference)	330,335	26535	8.03	1.00
Risk factors	All n = 858,404	With PPH n = 38,270		
Deliveries with 0 risk factors	373,915	21,770	5.82	1.00
Deliveries with at least 1 risk factor	217,480	24,720	11.37	1.95
Deliveries with at least 2 risk factors	153,425	18,215	11.87	2.04
Comorbidities	All n = 858,404	With PPH n = 38,270		
Deliveries with 0 comorbidity	216,135	13,605	6.29	1.00
Deliveries with at least 1 comorbidity	359,805	32,855	9.13	1.45
Deliveries with at least 2 comorbidities	239,405	23,030	9.62	1.53

**Table 3:** Comorbidity burden in postpartum hemorrhage (PPH). The prevalence of comorbidities in PPH deliveries (blue) and the reciprocal prevalence of PPH in comorbid conditions (orange).

Causes	PPH n = 69,745
Atony	53,325 (76.46%)
Trauma	13,515 (19.38%)
Tissue-related	21,945 (31.47%)
Thrombin-induced	1,835 (2.63%)
Interventions	PPH n = 69,745
Hysterectomy	1,280 (2.40%)
Surgical procedures (including hysterectomy)	8,920 (12.79%)
Manipulative procedures	11,000 (15.77%)
Blood transfusion	6,000 (8.60%)
Medications	37,720 (54.08%)

## Data Availability

The datasets generated and analyzed during the current study are not publicly available due to patient privacy regulations but are available from the corresponding authors on reasonable request.

## Code availability

The underlying code for this study is not publicly available but may be made available to qualified researchers on reasonable request from the corresponding authors.
